# Supplementary material for: Cannabidiol increases gramicidin current in human embryonic kidney cells: An observational study
Source: PLoS One. 2022 Aug 1;17(8):e0271801. doi: 10.1371/journal.pone.0271801 (PMC9342711; doi:10.1371/journal.pone.0271801)
Supplement: S1 Fig — (A) Shows the time course of experiment at high sodium and (B) at low sodium concentrations. (DOCX) [file pone.0271801.s001.docx]

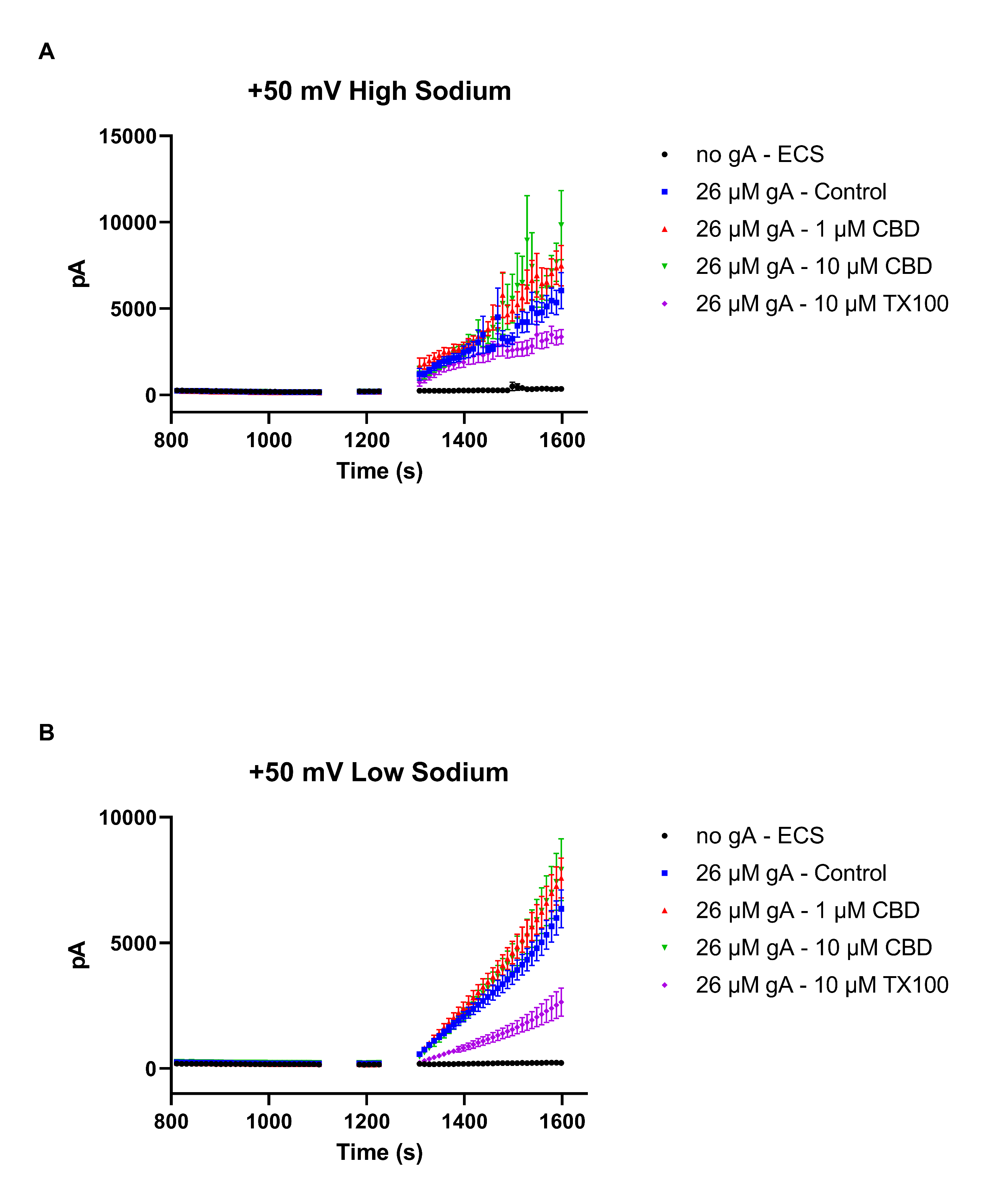


**Figure S1 – Time course of experiment.**

(A) Shows the time course of experiment at high sodium and (B) at low sodium concentrations.
