# Supplementary material for: Cannabidiol increases gramicidin current in human embryonic kidney cells: An observational study
Source: PLoS One. 2022 Aug 1;17(8):e0271801. doi: 10.1371/journal.pone.0271801 (PMC9342711; doi:10.1371/journal.pone.0271801)
Supplement: Attachment — Submitted filename: This manuscript by Ghovanloo Goodchild and Ruben describes the effects of CBD on gramicidin channels incorporated in HEK293 cells.docx [file pone.0271801.s002.docx]

This manuscript by Ghovanloo Goodchild and Ruben describes the effects of CBD on gramicidin channels incorporated in HEK293 cells, using patch clamp.

Major comments

The description of the methods appears insufficient. In particular, application of gramicidin on only one side of the membrane would be difficult to compare to gramicidin added in the tiny amount used when added to both side of lipid bilayers. Did the authors thought of using a low concentration of gramicidin in the pipette to let the two sides bring a small amount of gramicidin as in the bilayer experiments of Andersen’s lab? How much was the stock in DMSO? How much DMSO was added to get to 26 μM?

It would be useful to see the full temporal course of the experiment. This is because the actions of gramicidin on biological membranes tend to take long to equilibrate when gramicidin is added on only one side (although this would depend on the concentration used and the biological membrane being considered). This will demonstrate that the drugs were always added only after the current is completely stable can the drugs be added. If this is not possible, at least give the time course of increase in conductance after application of gramicidin and how long after gramicidin application were the drugs added, and how long was the waiting time before measurements in the presence of drug.

I believe the large concentrations of gramicidin required to form pores in the whole cell are the reason why opposite than expected results are observed with triton. It would be good to discuss the differences between what is observed in HEK cells and what has been described in lipid bilayers and the possibility that at these massive concentrations part of the gramicidin channels are not as well behaved as in the bilayers. I think that it is good that the authors have the results in both systems. Perhaps what happens is that large part of the channels are not in the conformation normally seen at the low concentrations used in artificial lipid bilayers.

The gramicidin monomer/dimer association dissociation is not a conformational change. The channel associates and dissociates and the energetic cost for this only comes from the membrane deformation, please correct that statement.

About the discussion

I don’t think this results suggest that there is a different mechanism, I think this suggests that the channels formed by gramicidin are not the well behaved channels seen in artificial bilayers. This will require reviewing literature about different structures of gramicidin channels can adopt (I won’t dare to say this is a double stranded gramicidin, see Andersen et al NSB, 1999), but clearly gramicidin channel in artificial membranes form at concentrations of 10 nM not the 26 μM used in this work or to increase permeability in biological membranes. The possibility of the channel adopting strange conformations should be considered here, even if its cation selectivity remains.

As presented more than indicating that CBD acts through a different mechanism than bilayer modification, the data indicates that triton may be interacting directly with the gramicidin channel. I say this, because the observation with CBD is an increase in current, as expected from the GFA. The effect of triton is not.

Minor comments.

3^rd^ line of the discussion, please correct the statement on bilateral modification to

These findings indicate that CBD is a modifier of the bilayer physical properties at the tested concentrations of 1-30 μM ...

The room temperature appears to be very high (27C), I am just confirming this is not a typo occurring twice.
